# Essential Oil Extraction, Chemical Analysis and Anti-*Candida* Activity of *Calamintha nepeta* (L.) Savi subsp. *glandulosa* (Req.) Ball—New Approaches

**DOI:** 10.3390/molecules22020203

**Published:** 2017-01-26

**Authors:** Mijat Božović, Stefania Garzoli, Manuela Sabatino, Federico Pepi, Anna Baldisserotto, Elisa Andreotti, Carlo Romagnoli, Antonello Mai, Stefano Manfredini, Rino Ragno

**Affiliations:** 1 Rome Center for Molecular Design, Sapienza University, P.le Aldo Moro 5, 00185 Rome, Italy; mijat.bozovic@uniroma1.it (M.B.); manuela.sabatino@uniroma1.it (M.S.); 2 Department of Drug Chemistry and Technology, Sapienza University, P.le Aldo Moro 5, 00185 Rome, Italy; stefania.garzoli@uniroma1.it (S.G.); federico.pepi@uniroma1.it (F.P.); antonello.mai@uniroma1.it (A.M.); 3 Department of Life Sciences and Biotechnology, School of Pharmacy and Heath Products, University of Ferrara, Via L. Borsari 46, 44121 Ferrara, Italy; anna.baldisserotto@unife.it; 4 Department of Life Sciences, University of Modena and Reggio Emilia, Viale Caduti in Guerra 127, 41121 Modena, Italy; elisa.andreotti@unimo.it (E.A.); carlo.romagnoli@unimo.it (C.R.); 5 Alchemical Dynamics s.r.l., 00125 Rome, Italy

**Keywords:** *Calamintha nepeta* (L.) Savi subsp. *glandulosa* (Req.) Ball, 24-h steam distillation, GC/MS, essential oils, anti-*Candida* activity, pulegone, menthone

## Abstract

A comprehensive study on essential oils extracted from different *Calamintha nepeta* (L.) Savi subsp. *glandulosa* (Req.) Ball samples from Tarquinia (Italy) is reported. In this study, the 24-h steam distillation procedure for essential oil preparation, in terms of different harvesting and extraction times, was applied. The Gas chromatography–mass spectrometry (GC/MS) analysis showed that *C. nepeta* (L.) Savi subsp. *glandulosa* (Req.) Ball essential oils from Tarquinia belong to the pulegone-rich chemotype. The analysis of 44 samples revealed that along with pulegone, some other chemicals may participate in exerting the related antifungal activity. The results indicated that for higher activity, the essential oils should be produced with at least a 6-h steam distillation process. Even though it is not so dependent on the period of harvesting, it could be recommended not to harvest the plant in the fruiting stage, since no significant antifungal effect was shown. The maximum essential oil yield was obtained in August, with the highest pulegone percentage. To obtain the oil with a higher content of menthone, September and October should be considered as the optimal periods. Regarding the extraction duration, vegetative stage material gives the oil in the first 3 h, while material from the reproductive phase should be extracted at least at 6 or even 12 h.

## 1. Introduction

Plant essential oils (EOs) are usually complex mixtures of natural compounds, both polar and nonpolar [1]. They are principally composed of terpenoids and their oxygenated derivatives. Possessing antioxidant and antimicrobial activities, EOs serve as natural additives in foods and food products [2]. Known for their antiseptic (i.e., bactericidal, virucidal and fungicidal), medicinal properties and their fragrance, they are used in embalmment, preservation of foods and as antimicrobial, analgesic, sedative, anti-inflammatory, spasmolytic and local anesthetic remedies [3].

Indeed, many EOs and their ingredients have been proven to exhibit a wide range of biological activities that is even difficult to comprehensively report. In particular, antimicrobial properties of EOs are subject of continuous investigation both in vitro [4,5] and in vivo [6,7] against a wide range of pathogenic bacteria and fungi.

According to literature, EOs are mainly obtained by hydro- or steam distillation apparatus for 2–4 h (optionally 5). On the other hand, most of the data are related to some specific period of harvesting, e.g., flowering or fruiting periods. A comprehensive study on the EO, considering both extraction time and period of harvesting, from wild *Mentha suaveolens* Ehrh. (MS) has been reported recently [8]. Analogously, as in the MS study, we report herein a detailed study on *C. nepeta* (L.) Savi subsp. *glandulosa* (Req.) Ball (CG) EOs from Tarquinia (TEOCG), its chemical composition and the related antifungal activity.

### Taxonomic Characterization and Uses of C. nepeta (L.) Savi and Its Subspecies

The genus *Calamintha* Miller includes aromatic plants belonging to the Lamiaceae family, well represented and widespread all around the Mediterranean region. These are medium to large size erect herbaceous perennials, sometimes woody at the base, represented by five polymorphic species in the European flora [9]. *C. nepeta* (L.) Savi (CN) is a bushy, rhizomatous herb similar to the common mint in its morphology and characteristic fragrance [10]. It includes two subspecies: *nepeta* (CNN) and *glandulosa* (CG), with the main differences in the number of flowers in cymes and leaf shape and size.

**Figure 1:**
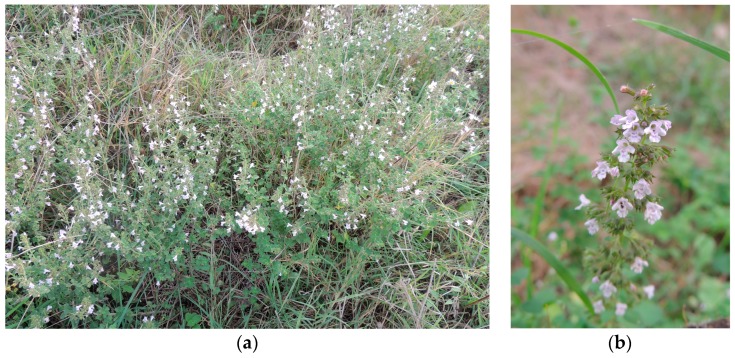
(**a**) *C. nepeta* (L.) Savi subsp. *glandulosa* (Req.) Ball (CG) in its natural habitat in Tarquinia countryside; (**b**) CG, close-up (photo: Mijat Božović).

The subspecies CG, which was the subject of this investigation, grows up to 80 cm. It is sparsely to densely pubescent, with leaves broadly ovate, obtuse, subentire or shallowly to deeply crenate-serrate with up to five teeth on each side [9] that are very fragrant when crushed. It typically forms a dense foliage usually found on rocky sites, dry meadows and abandoned places. Tiny, tubular, two-lipped, lilac to white flowers appear in axillary spikes (cymes 5- to 15-flowered) (Figure 1). It usually grows in summer (June/July), blooms during late summer followed by fruiting in autumn. Then it becomes dormant in the winter months, and re-blossoms in summer.

A careful literature survey showed plenty of data, but most of them are related to CNN, or the material was not described in terms of subspecies (determined as CN). The latter occurrence makes it particularly difficult to compare the existing data and to draw conclusions, taking into account the possibility of incomplete or even incorrect determination of material. Lastly, it can be said that these two subspecies share most of the uses and applications, since they are utilized in traditional medicine in different countries. Thus, CN is well-known for its medicinal uses as a stimulant, tonic, antiseptic and antispasmodic [11,12,13,14]. The chemical composition of its EO (TEOCG) has been thoroughly investigated [11,12,13,15,16,17,18], as well as its antioxidant [19,20], antimicrobial [14,21,22,23] and anti-inflammatory activities [14]. In the traditional medicine of different countries in the world, CN has also been widely used against insomnia, depression, convulsion and cramps [24] and for the treatment of respiratory and gastro-enteric diseases [14]. In several parts of Sicily, it is used for the disinfection and healing of wounds [21]. Its use against gout, expectorant and an external application of the leaves against hip pains, has been reported [14,25]. It is also used as a spice in Italian cuisine where it is called *mentuccia* or *nipitella*.

Several studies on TEOCG chemical composition were reported revealing different chemotypes in which the major components generally belong to the C-3 oxygenated *p*-menthanes [19] and can be grouped into three types of oils. The first and most popular chemotype is characterized by pulegone (PUL) as a major component, associated with menthone (MEN). It has been found wild in Italy [21,26], France [11], Turkey [12,13,14], Croatia [27] and Montenegro [22]. The second type is still characterized by PUL as a major component but associated with piperitenone. This chemotype has been reported in the material from Turkey [28], Greece [29] and Montenegro [22]. The third one can be considered as a piperitone oxide type, specific for the CG growing in Greece [30], or the piperitone oxide/piperitenone oxide one that has been noticed for the material from Turkey [13,31] and Belgium [32]. A piperitone/piperitenone variant of this type was only reported for some Croatian material [19].

## 2. Results and Discussion

### 2.1. EO Extraction

Fresh aerial parts of CG were subjected to steam distillation and the oil was collected at various times (1, 2, 3, 6, 12 and 24 h) during four successive months (see Material and Methods Section). The EO yield varied in function of both the harvesting period and the extraction intervals. The results showed different amounts of each fraction, but usually the main part of EOs was obtained in the first three (July harvest/August harvest) or six hours (September harvest/October harvest).

**Figure 2:**
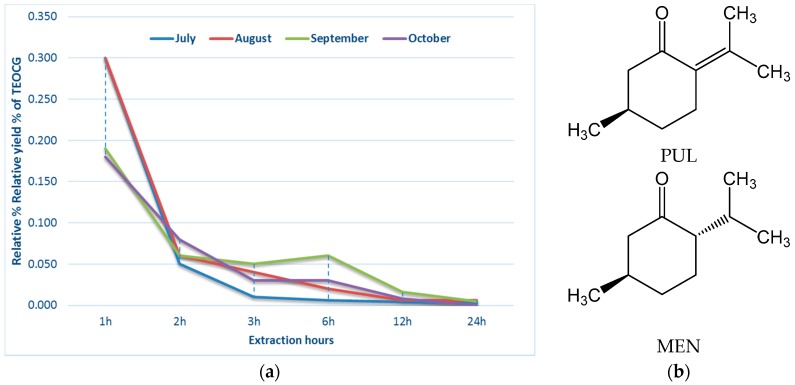
(**a**) Yield curves for CG; (**b**) chemical structures of pulegone (PUL) and menthone (MEN).

In the July harvest, the EO was almost completely extracted in the first two hours. Higher amounts of EO were extracted in the August harvest, likely due to higher temperature and other environmental conditions. Blooming material from the September harvest was characterized by high EO yield, not only in the first three fractions, but also between the third and sixth hours of extraction. The last one, October harvest material, was mainly in the fruiting phase and was characterized by the lowest yields (Figure 2a, Table 1).

### 2.2. GC/MS Analysis

The GC/MS analysis of the 44 TEOCG samples highlighted the presence of 39 different chemical constituents, having different concentrations in the various fractions (Table 2, Table 3, Table 4 and Table 5 and S1–S4 of Supplementary Materials). In general, PUL (**36**) was the most abundant component. Based on the extraction time in the July and August harvest, PUL percentage reached the maximum during the first three hours (J2h, A2h) whereas it diminished during the last three extractions. In the reproductive period of September and October, PUL percentage reached the maximum between the third and sixth (S6h) or twelfth hours (O12h). In all cases, PUL never disappeared and was present in smaller amounts in the last three fractions. It was particularly abundant in the August (84.7%) and July harvest (77.7%) samples. These findings are in compliance with previous reports [11,12,13,14,17,21,22,26,28,29,31,32,33,34]. PUL is a monoterpene ketone (Figure 2b) with a fresh odor reminiscent of mint, found in many Lamiaceae species. It was first isolated from the EO of *Mentha pulegium* L. from which its name was derived. This colorless liquid is very low soluble in water (less than 1%), but miscible with organic solvents such as ethanol, diethyl ether and chloroform. Biochemically, PUL is derived from terpinolene through piperitenone, and it is also the precursor of MEN, isomenthone and isopulegone [35] and is usually encountered in combination with one or more of the mentioned compounds [36].

The appearance of other constituents is related to the extraction time, but some of them are always present in significant amounts. For instance, MEN (**26**) was present in each fraction (except J24h) and each month. Its percentage in the July harvest was important only in the first fraction (J1h), while in the August harvest its presence is significant also in the next two fractions (up to three hours of the extraction process). September and October harvest materials were characterized by a substantial increase of MEN (up to 35.4% in O1h), particularly in the first three hours, displaying almost 10 times the amount obtained from the plant collected in July and August. The substantial presence of MEN (Figure 2b) in the CG oils has already been reported [12,13,14,21,22,34], sometimes up to 60% [11]. According to results of the GC/MS analysis, samples from Tarquinia clearly belong to the PUL/MEN chemotype. However, the PUL/MEN ratio is influenced by the growth stage of the plant, with the greatest increase of MEN% during the fruiting period (October). This evolution of EO composition with the growth of the plant is somehow in good compliance with the report on EO from Corsica [11].

Crysanthenone (CRY, **17**) gradually increases its amount with the extraction time. In the July harvest, its percentage was from 4.4% to 20.3% in the first three hours (J1h–J3h) up to 33.9% in the twelfth extraction hour (J12h). The same compound in the August harvest increased from 2.6% to only 9% in the first three fractions (A1h–A3h) and then rapidly increased two times between the third and sixth hours up to 29.5% in the last fraction (A24h). It can be noticed that CRY is present at higher percentage in the July harvest and August harvest, than in flowering and fruiting periods when the maximum was 18.6% and 13.4% (S24h and O24h). This chemical compound was not reported to be an important constituent of CG oils. There are only few reports about its occurrence [33] and none is even close to the percentage found in TEOCG, indicating that the Tarquinia plants belong to a PUL/MEN chemotype variant. Considering the average distillation process duration (usually up to two–three hours) in most cases, CRY un-detection is not surprising, as it becomes significant between 3 and 6 h of extraction. On the other hand, this compound is believed to be dependent on undergoing thermal and hydrolytic reactions during the hydro distillation process [37], possibly explaining the percentage increase with the time of extraction.

Limonene (LIM, **19**) is the characteristic component of the first fraction (and exceptionally in the second) of each month (J1h, A1h, S1h and O1h), reaching the maximum percentage (13.6%) in the flowering material gathered in the September harvest as described in previous reports [11,13,14,19,21,22,31,32,33]. As a part of each CG chemotype, LIM could be considered as the common component, likely responsible for the plant fragrance [32]. Regarding other chemical components, some compounds are present only in particular fractions, such as methylisopulegone (**27**) in J24h (12.6%). Some others are characteristic of specific months: e.g., *p*-cymene (**7**) is present only in the August harvest, while germacrene D (**20**) and α-terpineol (**38**) are only found in the July harvest. Caryophyllene oxide (**14**) is missing only in the July harvest, while linalool (**24**) is not present in the EOs during reproductive periods. On the other hand, piperitenone (**30**) appears in the blooming period (August–September) and disappears in the October harvest, notably increased in amount in the last fractions (S6h, S12h and S24h). Piperitenone oxide (**31**) was detected only in the September and October harvest EOs, reaching the higher quantities in the fruiting period (O3h to O24h). These observations prove that some constituents are characterizing compounds in reproductive periods.

In general, the complexity of the TEOCG chemical composition was more or less the same in the first three months (from 19 to 22 compounds), and rapidly decreased in the October harvest with just 13 different chemical constituents.

In order to simulate continuous EO extraction, the 1, 2, 3, 6, 12 and 24 h fractions were combined to generate EOs as there would have been extracted in single runs of 2, 3, 6, 12 and 24 h (Supplementary Materials Tables S1–S4). The mixtures were prepared by hand and, taking into account the respective yields, reflect quite well the compositions of the original fractions, and were checked by GC/MS analyses for consistency. PUL was still the main characterizing compound, with MEN in the last two months. However, some deviations could be observed: the first mix from the October harvest (OM1, Table S4) is characterized by a very high percent of MEN (not so high in the original fractions) and quite a low percentage of PUL (in comparison with the original fractions). This occurrence could be explained by some mistake during the mixing process.

It can also be noticed that some compounds are missing in the mixtures, making them simpler than the original fractions. For instance, linalool (**24**), α-terpineol (**38**) and cinerolone (**15**) in the July harvest and caryophyllene oxide (**14**) in the October harvest.

### 2.3. Anti-Candida Activity

The in vitro antifungal activities of TEOCGs extracted at different times and in different months, against *Candida albicans* (ATCC 10231), are reported in Table 6. The anti-*Candida* efficacy was compared to that of miconazole (minimum inhibitory concentration (MIC) = 0.016 mg·mL^−1^), a well-known antifungal synthetic drug, and to that of solvent used to dilute the oils as blank (RPMI 1640 supplemented with Tween 80) that has no activity against *Candida*. The results are representative of two independent experiments (24 and 48 h of incubation) arranged in triplicate. With few exceptions, the MIC of this strain ranged from 6.24 mg·mL^−1^ to 12.48 mg·mL^−1^ for TEOCG extracted in the July and October harvest, and from 3.12 to 12.48 mg·mL^−1^ for TEOCG extracted in the August and September harvest. Notably, some extracts showed an interesting and significant antifungal activity with MIC ranging from 0.78 to 1.56 mg·mL^−1^.

Usually, the third fraction (between the second and third hours of extraction) showed good activity (J3h, A3h) or even the fourth one (between the third and sixth hours) in the case of the September harvest (S6h). In the case of mixtures, significant activity was detected for the third or fourth mix (JM3, AM3, SM3 and SM4). On the contrary, the October harvest was lacking any significant activity.

A survey of the available literature highlighted that most of the data about antimicrobial activity are related to CN [21,26,38], but not to the subspecies investigated in this paper. To the best of our knowledge, the only data reported for CG was from the Montenegrin material [22] and from Turkey [23,39]. The EO was screened against the following bacteria: *Escherichia coli*, *Staphylococcus aureus*, *Salmonella enteritidis*, *Bacillus subtilis*, *Pseudomonas aeruginosa* and fungi *Aspergillus niger*. All the microorganisms (except *S. enteritidis*) were found to be susceptible, specifically *A. niger* [22]. Antimicrobial activity of the Turkish sample was evaluated, using the disc diffusion method, and the study showed that all the bacteria (particularly *B. subtilis*, *Staphylococcus epidermidis*, *Stenotrophomonas maltophilia*) and *C. albicans* were affected by the EO. In the study, the occurrence was explained by the high percentage of PUL [23]. In addition, different CG extracts and fractions were investigated against Gram(+) *B. subtilis* and Gram(−) *E. coli* and *Salmonella typhimurium* [39]. The analysis showed better activity of the water extract than the methanol one, whereas the fractions prepared had greater bactericidal efficacy, especially the dichloromethane one. However, methanolic extract and the ethyl acetate fraction failed to inhibit all tested bacteria.

The contribution of some pure constituents to the EO effectiveness has also been evaluated [21]. The main constituents of TEOCG were analyzed, and the only one endowed with antibacterial effectiveness was PUL. The authors indicated that the effectiveness of the essence was almost exclusively due to that constituent [21]. The results indicate that PUL is likely not the only chemical component responsible for the antifungal activities, although it is always the dominant one, on the other hand, this is just an observation as advanced chemometric and statistical analyses need to be carried out. Looking at Tables S1–S4, it is evident that other minor constituents are capable of microbiological activity as well and might be involved in the inhibition process with some synergistic mechanism resembling the one recently described [8]. This is in compliance with the phytocomplex hypothesis discussed elsewhere [40,41]. Further analysis of data indicates the lack of any significant correlation between the antimicrobial activity and the vegetative stage of the plant (Table 6). However, this is in contrast with some literature data which indicate the highest antimicrobial efficacy of EOs obtained during the blooming period of a plant [42,43,44].

## 3. Materials and Methods

### 3.1. Plant Material

Aerial parts of CG were collected in a wild area about 15 km from the city of Tarquinia (Viterbo, Italy), during 2015. Material was collected in summer and early autumn periods of the year and monitored for 4 months, from July to October thus covering before-, within- and post-flowering periods. Taxonomic identifications of the species were conducted according to the official European flora [9] and the national Italian one [45].

### 3.2. EO Extraction

Similarly, as previously reported [8] fresh plant material (2.5 kg) was subjected to steam distillation separating EOs at an interval time of 1, 2, 3, 6, 12 and 24 h and worked out to furnish six fractions of yellow paled EOs. In addition, EOs mixtures were prepared by mixing each fraction as follows: M1 (1 + 2 h of extraction), M2 (1 + 2 + 3 h), M3 (1 + 2 + 3 + 6 h), M4 (1 + 2 + 3 + 6 + 12 h) and M5 (1 + 2 + 3 + 6 + 12 + 24 h). The mixtures were prepared by adding different amounts of diethyl ether to each EO fraction up to 10 mL in total (e.g., 7 mL of diethyl ether to 3 mL of the EO). Desired oily mixes were obtained by combining 1 mL of each ether-EO solution and then leaving ether to evaporate. This procedure allowed the study of EO extraction by obtaining its fractions after 2, 3, 6, 12 and 24 h, within parallel procedures which were not interrupted.

### 3.3. EO Analysis

The gas chromatographic/mass spectrometric (GC/MS analysis was carried out with a GC-MS and flame ionization detector (FID) as previously reported [8]. The analyses were carried out twice showing reproducible results.

### 3.4. Antimicrobial Assay

As previously reported, the MIC was determined by the micro-broth dilution method (microsterile plate) according to the Clinical and Laboratory Standards Institute/National Committee for Clinical Laboratory Standards (CLSI/NCCLS) Approved Standard M27-A3, 2008 [NCCLS]. Miconazole (0.5 mg·mL^−1^), used as positive control, was prepared by dissolving the agent in endotoxin free water. EO solutions (100 mg·mL^−1^) were prepared in RPMI 1640. Shortly, to determine the MIC of TEOCG extracted at different times and in different months, or miconazole, RPMI-1640 supplemented with 3-(N-morpholino)propanesulfonic acid (MOPS) at pH 7 was used. TEOCG was diluted in RPMI-1640 supplemented with Tween 80 (final concentration of 0.001% *v*/*v*). RPMI-1640 supplemented with Tween 80 was used as blank. Dilutions, 11 increasing concentrations, ranging from 0.012 to 12.48 mg·mL^−1^ of the EO, were prepared in 96-well plates. The inoculum size was about 2.5 × 10^3^ cells·mL^−1^. The plates were incubated at 30 °C for 24–48 h.

### 3.5. Statistical Evaluations

Relative standard deviations and statistical significance (Student’s *t* test; *p* ≤ 0.05) were given where appropriate for all data collected. One-way ANOVA and Least Significant Difference (LSD) post hoc Tukey’s honest significant difference test were used for comparing the bioactive effects of different samples. All computations were made using the statistical software STATISTICA 6.0 (StatSoft Italia s.r.l., Padova, Italy).

## 4. Conclusions

Since the very beginning, plants have provided human beings with a source of inspiration for novel medicines. Considering the increased interest in natural substances, the deepening of the knowledge of the potential of different aromatic plants can lead to important results in the discovery of both sustainable and effective drug treatment. In line with our previous studies on MS [8], a 24-h extraction procedure was applied to CG in terms of a different harvesting period (four months) and extraction duration. In this study, we investigated the chemical composition of 44 TEOCG samples, showing that the PUL/MEN chemotype was represented in Tarquinia, but the ratio varied greatly according to the growth phase of the plant. The MEN percentage increased at the blooming period, while the PUL decreased. The CRY amount increased with the longer distillation process. This could be considered as the characteristic of TEOCG or the result of transformation during the process. Antifungal activities of the samples have also been investigated, showing the significant efficacy of some samples. Taken together, obtained results point to synergistic effects of all the constituents of the EO, and that PUL cannot be considered as the only responsible constituent. It can be speculated that some components could have a positive and additive biological role, as well as possible anti-synergism with the most dominant EO ingredients.

In relation to the biological activity, the period of extraction seems to be of importance since great activities were shown by EOs collected between the third and sixth hours. Although the harvesting period seems not to be important, EO from the fruiting stage showed the lowest anti-*Candida* activity. Consideration should also be given to the EO production yield or the chemical composition, and harvesting in August led to the highest EO production, as well as PUL percentage. On the other hand, the main part of EOs was extracted in the first three (July harvest/August harvest) or six hours (September harvest/October harvest). Based on the extraction time in the July and August harvests, PUL percentage reached the maximum during the first three hours (J2h, A2h) whereas it diminished during the last three extraction phases. In the reproductive period of September and October, the situation was different and the PUL percentage maximum was between the third and sixth (S6h) or twelfth hours (O12h).

Further investigation of other biological activities will be carried out in due course.

## Figures and Tables

**Table 1:** Relative yield % of TEOCG over time.

h ^1^	July ^2^	August	September	October
1	0.300	0.300	0.190	0.180
2	0.350	0.360	0.250	0.260
3	0.360	0.400	0.300	0.290
6	0.366	0.420	0.360	0.320
12	0.370	0.426	0.376	0.328
24	0.373	0.432	0.381	0.328

^1^ extraction hour; ^2^ harvesting month.

**Table 2:** Chemical composition (%) of TEOCG extracted in July.

# ^1^	Name	Sample ^2^					
		J1h	J2h	J3h	J6h	J12h	J24h
**4**	3-octanol	2.2 ± 0.14	0.4 ± 0.03	0.3 ± 0.02	0.4 ± 0.03	0.5 ± 0.03	-
**5**	terpinen-4-ol	0.6 ± 0.04	0.5 ± 0.03	0.4 ± 0.02	0.4 ± 0.02	0.4 ± 0.02	-
**15**	cinerolone	-	-	-	2.9 ± 0.21	5.8 ± 0.42	-
**17**	crysanthenone	4.4 ± 0.41	10.5 ± 0.98	20.3 ± 1.89	22.7 ± 2.11	33.9 ± 3.15	27.3 ± 2.54
**18**	δ-cadinene	-	-	-	0.6 ± 0.04	0.8 ± 0.05	2.4 ± 0.16
**19**	limonene	5.9 ± 0.57	0.6 ± 0.06	0.2 ± 0.02	0.1 ± 0.01	0.1 ± 0.01	-
**20**	germacrene D	-	-	1.5 ± 0.08	2.7 ± 0.14	0.8 ±0.04	-
**21**	isocaryophyllene	0.3 ± 0.02	-	1.3 ± 0.11	2.9 ± 0.24	2.3 ± 0.19	3.8 ± 0.36
**22**	isopiperitenone	-	-	-	-	-	2.2 ± 0.19
**23**	isopulegone	0.6 ± 0.05	0.5 ± 0.05	0.5 ± 0.05	0.5 ± 0.05	0.4 ± 0.04	-
**24**	linalool	0.3 ± 0.02	-	0.2 ± 0.01	0.4 ± 0.02	0.5 ± 0.03	-
**26**	menthone	3.1 ± 0.25	0.8 ± 0.06	0.6 ± 0.05	0.6 ± 0.05	0.5 ± 0.04	-
**27**	methylisopulegone	-	-	-	-	-	12.6 ± 0.76
**28**	myrcene	0.4 ± 0.03	-	-	-	-	-
**29**	*p*-cymen-8-ol	-	-	-	0.7 ± 0.06	1.5 ± 0.13	2.2 ± 0.19
**33**	*p*-mentha-1,8-dien-3-one	-	0.6 ± 0.03	0.7 ± 0.04	1.2 ± 0.07	2.0 ± 0.12	-
**34**	*p*-menthene	-	-	-	0.2 ± 0.02	-	-
**36**	pulegone	76.8 ± 5.91	77.7 ± 5.98	64.3 ± 4.95	53.2 ± 4.09	41.1 ± 3.16	37.7 ± 2.90
**37**	sabinene	0.6 ± 0.05	-	-	-	-	-
**38**	α-terpineol	0.3 ± 0.02	0.5 ± 0.03	0.7 ± 0.04	0.8 ± 0.05	1.2 ± 0.07	-
**39**	*trans*-*p*-mentha-2,8-dienol	-	-	-	0.2 ± 0.02	0.1 ± 0.01	-
Unidentified compounds		4.5 ± 0.25	7.9 ± 0.44	9.0 ± 0.50	9.5 ± 0.53	8.1 ± 0.45	11.8 ± 0.66

^1^ # indicate the compound identification number; ^2^ Samples’ names were obtained by merging the first letter of the month and the extraction time as reported in Table 1. All compounds are not listed in order of elution.

**Table 3:** Chemical composition (%) of TEOCG extracted in August.

# ^1^	Name	Sample ^2^					
		A1h	A2h	A3h	A6h	A12h	A24h
**4**	3-octanol	1.6 ± 0.15	0.4 ± 0.04	0.2 ± 0.02	0.3 ± 0.03	0.5 ± 0.05	0.5 ± 0.05
**5**	terpinen-4-ol	0.4 ± 0.04	0.4 ± 0.04	0.5 ± 0.05	0.5 ± 0.05	0.7 ± 0.06	0.6 ± 0.05
**7**	*p*-cymene	0.5 ± 0.05	0.4 ± 0.03	0.3 ± 0.03	0.5 ± 0.05	0.3 ± 0.03	-
**9**	β-myrcene	0.5 ± 0.03	-	-	-	-	-
**12**	β-terpinene	0.8 ± 0.05	0.1 ± 0.01	-	-	-	-
**14**	caryophyllene oxide	-	-	-	1.2 ± 0.09	1.8 ± 0.13	-
**17**	crysanthenone	2.6 ± 0.25	5.2 ± 0.49	9.0 ± 0.86	18.4 ± 1.77	24.0 ± 2.30	29.5 ± 2.83
**18**	δ-cadinene	-	-	-	-	0.5 ± 0.04	1.1 ± 0.08
**19**	limonene	7.5 ± 0.46	1.0 ± 0.06	0.6 ± 0.04	0.6 ± 0.04	0.2 ± 0.01	-
**21**	isocaryophyllene	-	-	-	-	1.3 ± 0.09	2.4 ± 0.17
**23**	isopulegone	0.6 ± 0.05	0.6 ± 0.05	0.6 ± 0.05	0.6 ± 0.05	0.8 ± 0.06	0.7 ± 0.05
**24**	linalool	0.4 ± 0.03	0.2 ± 0.01	-	0.2 ± 0.01	0.4 ± 0.03	0.3 ± 0.02
**25**	menthol	0.4 ± 0.03	0.5 ± 0.03	0.5 ± 0.03	0.5 ± 0.03	0.4 ± 0.03	0.4 ± 0.03
**26**	menthone	3.9 ± 0.37	2.1 ± 0.19	1.0 ± 0.09	0.8 ± 0.08	0.7 ± 0.07	0.7 ± 0.07
**30**	piperitenone	-	-	-	0.5 ± 0.03	0.8 ± 0.04	0.9 ± 0.05
**33**	*p*-menth-1,8-dien-3-one	-	-	0.4 ± 0.03	0.6 ± 0.04	1.0 ± 0.06	1.9 ± 0.12
**32**	*p*-menth-1-en-8-ol	-	0.5 ± 0.03	0.6 ± 0.04	1.0 ± 0.07	1.6 ± 0.11	2.7 ± 0.19
**36**	pulegone	80.8 ± 5.49	84.7 ± 5.76	80.0 ± 5.44	66.0 ± 4.49	55.4 ± 3.77	49.9 ± 3.39
Unidentified compounds		0.0	3.9 ± 0.22	6.3 ± 0.36	8.3 ± 0.47	9.6 ± 0.55	8.4 ± 0.48

^1^ # indicate the compound identification number; ^2^ Samples’ names were obtained by merging the first letter of the month and the extraction time as reported in Table 1. All compounds are not listed in order of elution.

**Table 4:** Chemical composition (%) of TEOCG extracted in September.

# ^1^	Name	Sample ^2^					
		S1h	S2h	S3h	S6h	S12h	S24h
**2**	2-hydroxypiperitenone	-	-	-	-	-	1.1 ± 0.09
**4**	3-octanol	3.0 ± 0.21	1.7 ± 0.12	0.8 ± 0.05	-	-	-
**5**	terpinen-4-ol	0.7 ± 0.06	0.6 ± 0.05	0.7 ± 0.06	0.6 ± 0.05	0.9 ± 0.07	0.7 ± 0.06
**7**	*p*-cymene	0.8 ± 0.08	-	-	-	-	-
**9**	β-myrcene	0.6 ± 0.06	-	-	-	-	-
**11**	β-pinene	1.2 ± 0.08	-	-	-	-	-
**14**	caryophyllene oxide	0.3 ± 0.02	0.5 ± 0.03	0.6 ± 0.03	0.7 ± 0.04	1.6 ± 0.08	1.8 ± 0.09
**15**	cinerolone	-	-	-	-	-	2.9 ± 0.5
**17**	crysanthenone	1.3 ± 0.08	2.0 ± 0.13	3.4 ± 0.21	6.8 ± 0.43	13.6 ± 0.86	18.6 ± 1.17
**18**	δ-cadinene	-	-	-	-	-	1.1 ±0.08
**19**	limonene	13.6 ± 1.33	2.1 ± 0.20	1.2 ± 0.12	0.6 ± 0.06	0.7 ± 0.07	0.7 ± 0.07
**23**	isopulegone	1.1 ± 0.07	1.5 ± 0.09	1.3 ± 0.08	1.1 ± 0.07	1.1 ± 0.07	9.4 ± 0.58
**25**	menthol	1.3 ± 0.12	2.2 ± 0.20	1.9 ± 0.18	1.9 ± 0.18	1.7 ± 0.16	1.7 ± 0.16
**26**	menthone	20.3 ± 1.52	20.0 ± 1.50	11.2 ± 0.84	5.9 ± 0.44	4.1 ± 0.31	3.7 ± 0.28
**29**	*p*-cymen-8-ol	-	-	-	-	0.7 ± 0.06	1.5 ± 0.14
**30**	piperitenone	0.7 ± 0.06	1.5 ± 0.13	2.0 ± 0.17	3.1 ± 0.26	4.5 ± 0.38	3.9 ± 0.33
**31**	piperitenone oxide	1.3 ± 0.09	1.3 ± 0.09	0.8 ± 0.05	0.5 ± 0.03	-	-
**33**	*p*-menth-1,8-dien-3-one	-	-	-	-	0.9 ± 0.07	1.3 ± 0.09
**32**	*p*-menth-1-en-8-ol	0.2 ± 0.02	0.4 ± 0.03	0.5 ± 0.04	0.6 ± 0.05	1.1 ± 0.09	1.6 ± 0.14
**36**	pulegone	48.8 ± 4.39	62.5 ± 5.62	72.9 ± 6.56	74.9 ± 6.74	64.8 ± 5.83	43.2 ± 3.89
**37**	sabinene	1.4 ± 0.14	-	-	-	-	-
Unidentified compounds		3.4 ± 0.20	3.7 ± 0.22	2.7 ± 0.16	3.3 ± 0.19	4.3 ± 0.25	6.8 ± 0.40

^1^ # indicate the compound identification number; ^2^ Samples’ names were obtained by merging the first letter of the month and the extraction time as reported in Table 1. All compounds are not listed in order of elution.

**Table 5:** Chemical composition (%) of TEOCG extracted in October.

# ^1^	Name	Sample ^2^					
		O1h	O2h	O3h	O6h	O12h	O24h
**3**	3-metilcicloesanone	-	-	-	-	0.9 ± 0.06	1.9 ± 0.13
**4**	3-octanol	2.1 ± 0.19	1.0 ± 0.09	0.6 ± 0.05	0.3 ± 0.03	0.3 ± 0.03	0.7 ± 0.06
**5**	terpinen-4-ol	0.6 ± 0.03	0.7 ± 0.03	0.9 ± 0.04	0.9 ± 0.04	1.1 ± 0.05	0.8 ± 0.04
**14**	caryophyllene oxide	-	-	1.0 ± 0.06	1.2 ± 0.07	1.8 ± 0.11	1.7 ± 0.11
**17**	crysanthenone	1.3 ± 0.11	2.3 ± 0.19	3.3 ± 0.28	5.3 ± 0.44	5.4 ± 0.45	13.4 ± 1.12
**19**	limonene	9.2 ± 0.51	2.1 ± 0.11	0.6 ± 0.03	0.7 ± 0.04	0.8 ± 0.04	0.9 ± 0.05
**21**	iso-caryophyllene	0.4 ± 0.02	0.6 ± 0.04	0.5 ± 0.03	0.7 ± 0.04	2.0 ± 0.12	3.5 ± 0.21
**23**	isopulegone	1.0 ± 0.05	1.0 ± 0.05	1.2 ± 0.06	1.1 ± 0.06	1.3 ± 0.07	1.0 ± 0.05
**25**	menthol	4.3 ± 0.25	4.4 ± 0.25	6.3 ± 0.36	5.5 ± 0.32	5.3 ± 0.31	4.2 ± 0.24
**26**	menthone	35.4 ± 3.01	27.8 ± 2.36	23.6 ± 2.01	10.9 ± 0.93	7.0 ± 0.59	6.8 ± 0.58
**31**	piperitenone oxide	1.4 ± 0.14	1.9 ± 0.18	3.3 ± 0.32	3.6 ± 0.35	0.5 ± 0.05	5.2 ± 0.51
**32**	*p*-menth-1-en-8-ol	0.3 ± 0.02	0.3 ± 0.02	0.5 ± 0.03	0.8 ± 0.05	1.4 ± 0.09	2.3 ± 0.15
**36**	pulegone	42.5 ± 2.17	57.5 ± 2.93	53.3 ± 2.72	68.2 ± 3.48	68.8 ± 3.51	51.8 ± 2.64
Unidentified compounds		1.5 ± 0.13	0.4 ± 0.03	4.9 ± 0.43	0.8 ± 0.07	3.4 ± 0.29	5.8 ± 0.51

^1^ # indicate the compound identification number; ^2^ Samples’ names were obtained by merging the first letter of the month and the extraction time as reported in Table 1. Compounds are not listed in order of elution.

**Table 6:** Anti-*Candida albicans* activities of the 44 TEOCG samples. The antibacterial tests were carried out three times, and the average values were taken as the MICs.

Sample	MIC mg∙mL^−1^		PUL %	Sample	MIC mg∙mL^−1^		PUL %
	24 h	48 h			24 h	48 h	
J1h	6.24	6.24	76.8	S1h	6.24	12.48	48.8
J2h	6.24	12.48	77.7	S2h	6.24	12.48	62.5
J3h	0.78	6.24	64.3	S3h	3.12	12.48	72.9
J6h	na	na	53.2	S6h	1.56	6.24	74.9
J12h	12.48	12.48	41.1	S12h	3.12	12.48	64.8
J24h	na	na	37.7	S24h	12.48	na	43.2
JM1	12.48	12.48	72.8	SM1	3.12	12.48	52.4
JM2	12.48	na	78.6	SM2	3.12	3.12	55.6
JM3	1.56	6.24	73.4	SM3	1.56	6.24	60.8
JM4	6.24	6.24	77.6	SM4	0.78	6.24	53.6
JM5	12.48	12.48	76.2	SM5	3.12	6.24	55.6
A1h	3.12	12.48	80.8	O1h	6.24	12.48	42.5
A2h	3.12	6.24	84.7	O2h	6.24	12.48	57.5
A3h	1.56	3.12	80.0	O3h	6.24	12.48	53.3
A6h	3.12	6.24	66.0	O6h	12.48	12.48	68.2
A12h	6.24	na	55.4	O12h	12.48	12.48	68.8
A24h	6.24	12.48	49.9	O24h	na	na	51.8
AM1	3.12	6.24	75.7	OM1	6.24	12.48	10.1
AM2	0.78	3.12	78.8	OM2	6.24	12.48	50.5
AM3	3.12	6.24	76.9	OM3	12.48	12.48	52.0
AM4	3.12	3.12	76.4	OM4	12.48	12.48	49.8
AM5	12.48	12.48	79.4	OM5	6.24	12.48	52.1

## Supplementary Materials

The following are available online at: http://www.mdpi.com/1420-3049/22/2/203/s1, Table S1: Chemical composition (%) of TEOCG mixtures from July; Table S2: Chemical composition (%) of TEOCG mixtures from August; Table S3: Chemical composition (%) of TEOCG mixtures from September; Table S4: Chemical composition (%) of TEOCG mixtures from October.
