# Supplementary material for: Essential Oil Extraction, Chemical Analysis and Anti-Candida Activity of Calamintha nepeta (L.) Savi subsp. glandulosa (Req.) Ball—New Approaches
Source: Molecules. 2017 Jan 26;22(2):203. doi: 10.3390/molecules22020203 (PMC6155801; doi:10.3390/molecules22020203)
Supplement: Supplementary file 1 [file molecules-22-00203-s001.pdf]

Supplementary Materials: Essential Oil Extraction, Chemical Analysis and Anti-*Candida* Activity of *Calamintha nepeta* (L.) Savi subsp. *glandulosa* (Req.) Ball—New Approaches

Mijat Božović, Stefania Garzoli, Manuela Sabatino, Federico Pepi, Anna Baldisserotto, Elisa Andreotti, Carlo Romagnoli, Antonello Mai, Stefano Manfredini and Rino Ragno

Table S1. Chemical composition (%) of TEOCG mixtures from July.

# [1]	Name	Sample [2]				
		JM1	JM2	JM3	JM4	JM5
**4**	3-octanol	2.1 ± 0.18	1.8 ± 0.16	2.5 ± 0.22	2.0 ± 0.17	2.0 ± 0.17
**5**	terpinen-4-ol	6.4 ± 0.49	0.5 ± 0.04	0.5 ± 0.04	0.5 ± 0.04	0.7 ± 0.05
**8**	linalool	-	-	0.5 ± 0.04	-	-
**16**	cis-β-terpineol	0.5 ± 0.05	-	-	0.6 ± 0.06	0.6 ± 0.06
**17**	crysanthenone	4.6 ± 0.40	5.7 ± 0.49	5.2 ± 0.45	5.6 ± 0.49	5.7 ± 0.49
**19**	limonene	4.8 ± 0.37	4.4 ± 0.33	4.3 ± 0.33	1.9 ± 0.14	4.9 ± 0.38
**23**	isopulegone	0.7 ± 0.06	0.6 ± 0.05	0.8 ± 0.06	0.7 ± 0.06	0.7 ± 0.06
**26**	menthone	2.9 ± 0.24	2.6 ± 0.21	3.4 ± 0.28	2.7 ± 0.22	2.8 ± 0.23
**28**	myrcene	-	-	-	0.6 ± 0.04	0.5 ± 0.03
**36**	pulegone	72.8 ± 3.71	78.6 ± 4.0	73.4 ± 3.74	77.6 ± 3.96	76.2 ± 3.89
**37**	sabinene	-	-	-	0.7 ± 0.05	-
Unidentified compounds		5.2 ± 0.33	5.8 ± 0.37	9.4 ± 0.60	7.1 ± 0.45	5.9 ± 0.38

[1] # indicate the compound identification number; [2] Samples' names were obtained by merging the first letter of the month, the letter M (mixture) and the serial number of the mix. Compounds are not listed in order of elution.

Table S2. Chemical composition (%) of TEOCG mixtures from August.

# [1]	Name	Sample [2]				
		AM1	AM2	AM3	AM4	AM5
**4**	3-octanol	2.0 ± 0.18	1.6 ± 0.14	1.6 ± 0.14	1.6 ± 0.14	1.6 ± 0.14
**5**	terpinen-4-ol	0.4 ± 0.03	0.4 ± 0.03	0.5 ± 0.04	0.4 ± 0.03	0.4 ± 0.03
**1**	α-pinene	0.6 ± 0.05	0.6 ± 0.05	0.9 ± 0.07	0.8 ± 0.06	0.8 ± 0.06
**7**	*p*-cymene	0.5 ± 0.04	0.4 ± 0.03	0.5 ± 0.04	0.5 ± 0.04	0.5 ± 0.04
**14**	caryophyllene oxide	0.3 ± 0.01	0.6 ± 0.03	0.4 ± 0.02	0.5 ± 0.03	0.5 ± 0.03
**17**	crysanthenone	2.7 ± 0.18	3.0 ± 0.20	3.7 ± 0.25	3.9 ± 0.26	0.4 ± 0.03
**19**	limonene	6.2 ± 0.46	5.8 ± 0.43	5.6 ± 0.41	5.3 ± 0.39	5.3 ± 0.39
**23**	isopulegone	0.7 ± 0.05	0.6 ± 0.04	0.6 ± 0.04	0.7 ± 0.05	0.7 ± 0.05
**25**	menthol	0.5 ± 0.03	0.4 ± 0.02	0.4 ± 0.02	0.5 ± 0.03	0.5 ± 0.03
**26**	menthone	4.5 ± 0.37	3.7 ± 0.30	3.8 ± 0.31	3.8 ± 0.31	3.8 ± 0.31
**31**	piperitenone oxide	0.3 ± 0.02	0.2 ± 0.01	0.2 ± 0.01	0.2 ± 0.01	0.3 ± 0.02
**32**	*p*-menth-1-en-8-ol	0.4 ± 0.03	0.3 ± 0.03	0.4 ± 0.03	0.4 ± 0.03	0.5 ± 0.04
**35**	*p*-menthone	-	0.1 ± 0.006	-	-	-
**36**	pulegone	75.7 ± 6.28	78.8 ± 6.54	77.0 ± 6.39	76.4 ± 6.34	79.4 ± 6.59
Unidentified compounds		5.2 ± 0.29	3.5 ± 0.19	4.4 ± 0.24	5.0 ± 0.27	5.3 ± 0.29

[1] # indicate the compound identification number; [2] Samples' names were obtained by merging the first letter of the month, the letter M (mixture) and the serial number of the mix. Compounds are not listed in order of elution.

Table S3. Chemical composition (%) of TEOCG mixtures from September.

# [1]	Name	Sample [2]				
		**SM1**	**SM2**	**SM3**	**SM4**	**SM5**
**4**	3-octanol	2.6 ± 0.17	2.4 ± 0.16	2.1 ± 0.14	2.4 ± 0.16	2.2 ± 0.14
**5**	terpinen-4-ol	0.8 ± 0.06	0.9 ± 0.07	0.7 ± 0.05	1.2 ± 0.09	1.1 ± 0.08
**10**	β-ocimene	0.7 ± 0.04	0.7 ± 0.04	0.6 ± 0.03	0.7 ± 0.04	0.6 ± 0.03
**11**	β-pinene	0.8 ± 0.05	0.8 ± 0.05	0.7 ± 0.04	0.7 ± 0.04	0.6 ± 0.04
**14**	caryophyllene oxide	0.6 ± 0.05	0.5 ± 0.04	0.5 ± 0.04	0.8 ± 0.06	0.9 ± 0.07
**17**	crysanthenone	1.5 ± 0.12	1.7 ± 0.14	2.3 ± 0.19	3.6 ± 0.29	4.0 ± 0.32
**19**	limonene	10.2 ± 0.80	9.4 ± 0.74	8.3 ± 0.66	8.6 ± 0.68	7.3 ± 0.58
**23**	isopulegone	1.3 ± 0.11	1.3 ± 0.11	1.2 ± 0.10	1.5 ± 0.13	1.5 ± 0.13
**25**	menthol	1.6 ± 0.11	1.7 ± 0.11	1.6 ± 0.11	1.9 ± 0.13	2.0 ± 0.13
**26**	menthone	21.5 ± 1.91	19.7 ± 1.75	16.0 ± 1.42	18.4 ± 1.64	17.3 ± 1.54
**30**	piperitenone	1.0 ± 0.05	1.1 ± 0.06	1.5 ± 0.08	2.2 ± 0.12	2.2 ± 0.12
**31**	piperitenone oxide	1.4 ± 0.12	1.2 ± 0.10	1.0 ± 0.09	1.2 ± 0.10	1.2 ± 0.10
**36**	pulegone	52.4 ± 3.82	55.6 ± 4.06	60.8 ± 4.44	53.6 ± 3.91	55.6 ± 4.06
Unidentified compounds		3.6 ± 0.23	3.0 ± 0.19	2.7 ± 0.17	3.2 ± 0.21	3.5 ± 0.23

[1] # indicate the compound identification number; [2] Samples' names were obtained by merging the first letter of the month, the letter M (mixture) and the serial number of the mix. Some of the compounds found in these mixtures were not present in the original fractions: this is likely due to some contamination during the mixture preparation. Compounds are not listed in order of elution.

Table S4. Chemical composition (%) of TEOCG mixtures from October.

# [1]	Name	Sample [2]				
		**OM1**	**OM2**	**OM3**	**OM4**	**OM5**
**4**	3-octanol	0.6 ± 0.03	1.6 ± 0.09	1.6 ± 0.09	1.7 ± 0.09	1.5 ±0.08
**5**	terpinen-4-ol	0.2 ± 0.01	0.7 ± 0.05	0.7 ± 0.05	0.6 ± 0.04	0.7 ± 0.05
**6**	iso-caryophyllene	-	0.5 ± 0.04	0.6 ± 0.05	0.6 ± 0.05	0.7 ± 0.06
**17**	crysanthenone	0.1 ± 0.007	1.6 ± 0.11	1.8 ± 0.13	1.8 ± 0.13	2.0 ± 0.14
**19**	limonene	2.1 ± 0.11	6.2 ± 0.32	6.0 ± 0.31	5.6 ± 0.29	6.2 ± 0.32
**23**	isopulegone	0.3 ± 0.02	0.9 ± 0.06	0.9 ± 0.06	1.0 ± 0.07	0.9 ± 0.06
**25**	menthol	1.2 ± 0.11	3.7 ± 0.35	3.8 ± 0.36	4.0 ± 0.38	3.8 ± 0.36
**26**	menthone	84.6 ± 7.36	32.2 ± 2.80	30.6 ± 2.67	32.9 ± 2.86	30.0 ± 2.61
**30**	piperitenone	0.5 ± 0.05	-	1.7 ± 0.16	-	-
**31**	piperitenone oxide	-	1.5 ± 0.08	-	1.7 ± 0.09	1.7 ± 0.09
**36**	pulegone	10.1 ± 0.57	50.5 ± 2.88	52.0 ± 2.96	49.8 ± 2.84	52.1 ± 2.97
Unidentified compounds		0.3 ± 0.02	0.6 ± 0.04	0.3 ± 0.02	0.3 ± 0.02	0.4 ± 0.03

[1] # indicate the compound identification number; [2] Samples' names were obtained by merging the first letter of the month, the letter M (mixture) and the serial number of the mix. Compounds are not listed in order of elution.
